# Molecular Signatures of Response to Mecasermin in Children With Rett Syndrome

**DOI:** 10.3389/fnins.2022.868008

**Published:** 2022-05-31

**Authors:** Stephen Shovlin, Chloe Delepine, Lindsay Swanson, Snow Bach, Mustafa Sahin, Mriganka Sur, Walter E. Kaufmann, Daniela Tropea

**Affiliations:** ^1^Neuropsychiatric Genetics, Trinity Center for Health Sciences, Trinity Translational Medicine Institute, St James Hospital, Dublin, Ireland; ^2^Department of Brain and Cognitive Sciences, Simons Center for the Social Brain, Picower Institute for Learning and Memory, MIT, Cambridge, MA, United States; ^3^Department of Neurology, Rosamund Stone Zander Translational Neuroscience Center, Boston Children’s Hospital and Harvard Medical School, Boston, MA, United States; ^4^Department of Human Genetics, Emory University School of Medicine, Atlanta, GA, United States; ^5^Department of Neurology, Boston Children’s Hospital, Boston, MA, United States; ^6^Trinity College Institute of Neuroscience, Trinity College Dublin, Dublin, Ireland; ^7^FutureNeuro, The SFI Research Centre for Chronic and Rare Neurological Diseases, Dublin, Ireland

**Keywords:** Rett syndrome, mecasermin, insulin-like growth factor 1 (IGF1), methyl-CpG binding protein 2 (MECP2), biomarker

## Abstract

Rett syndrome (RTT) is a devastating neurodevelopmental disorder without effective treatments. Attempts at developing targetted therapies have been relatively unsuccessful, at least in part, because the genotypical and phenotypical variability of the disorder. Therefore, identification of biomarkers of response and patients’ stratification are high priorities. Administration of Insulin-like Growth Factor 1 (IGF-1) and related compounds leads to significant reversal of RTT-like symptoms in preclinical mouse models. However, improvements in corresponding clinical trials have not been consistent. A 20-weeks phase I open label trial of mecasermin (recombinant human IGF-1) in children with RTT demonstrated significant improvements in breathing phenotypes. However, a subsequent randomised controlled phase II trial did not show significant improvements in primary outcomes although two secondary clinical endpoints showed positive changes. To identify molecular biomarkers of response and surrogate endpoints, we used RNA sequencing to measure differential gene expression in whole blood samples of participants in the abovementioned phase I mecasermin trial. When all participants (*n* = 9) were analysed, gene expression was unchanged during the study (baseline vs. end of treatment, T0–T3). However, when participants were subclassified in terms of breathing phenotype improvement, specifically by their plethysmography-based apnoea index, individuals with moderate-severe apnoea and breathing improvement (Responder group) displayed significantly different transcript profiles compared to the other participants in the study (Mecasermin Study Reference group, MSR). Many of the differentially expressed genes are involved in the regulation of cell cycle processes and immune responses, as well as in IGF-1 signalling and breathing regulation. While the Responder group showed limited gene expression changes in response to mecasermin, the MSR group displayed marked differences in the expression of genes associated with inflammatory processes (e.g., neutrophil activation, complement activation) throughout the trial. Our analyses revealed gene expression profiles associated with severe breathing phenotype and its improvement after mecasermin administration in RTT, and suggest that inflammatory/immune pathways and IGF-1 signalling contribute to treatment response. Overall, these data support the notion that transcript profiles have potential as biomarkers of response to IGF-1 and related compounds.

## Introduction

Rett syndrome (RTT) is an X-linked neurodevelopmental disorder that affects predominantly females (∼1/9,000–1/10,000) (Kaufmann et al., 2016). The diagnosis of RTT is clinical, taking into account a phenotypic spectrum of severity. The four core diagnostic criteria that define classic/typical versus variant/atypical RTT are partial or complete loss of hand function, partial or complete loss of spoken language, impaired gait, and presence of repetitive hand movements termed hand stereotypies (Neul et al., 2010). All four criteria are required for the diagnosis of classic RTT, while atypical RTT is diagnosed when at least 2 of these 4 main criteria are present plus 5 of 11 supportive criteria (i.e., breathing disturbances, bruxism when awake, impaired sleep, tone abnormalities, peripheral vasomotor disturbances, scoliosis/kyphosis, growth retardation, small cold hands and feet, inappropriate laughing/screaming spells, diminished pain response, and intense eye communication) (Neul et al., 2010). These supportive criteria are also prevalent in classic RTT (Percy et al., 2010).

Rett syndrome is usually associated with a pathogenic mutation in the *methyl-CpG binding protein 2* (*MECP2)* gene, particularly in those individuals with the classic presentation (Neul et al., 2010). Genotype-phenotype correlations have led to identifying groups of *MECP2* mutations with different levels of severity (Cuddapah et al., 2014). *MECP2* encodes the methyl CpG-binding protein 2 (MeCP2), a chromatin binder and transcription regulator (Ip et al., 2018). Abnormal expression of MeCP2 results in impaired brain development and function associated with disruption in synaptic plasticity (Kaufmann et al., 2005; Asaka et al., 2006; Blackman et al., 2012). The discovery of mutations in *MECP2* (Amir et al., 1999) as the genetic abnormality associated with most cases of RTT, has led to the generation of mutant mouse models that replicate many features of the disorder (Chen et al., 2001; Guy et al., 2001; Nguyen et al., 2013; Ross et al., 2016). These mutant mouse models have become a valuable resource for the study of the molecular and cellular mechanisms underlying RTT, and for testing candidate treatments for the disorder (Katz et al., 2012). Management of RTT is mainly symptomatic (Kaufmann et al., 2016; Leonard et al., 2017); therefore, the discovery of disease-modifying therapies in models of RTT has become a priority in the field. Preclinical studies in mice have already identified several promising drugs, some of which have moved to clinical development (Tropea et al., 2009; Castro et al., 2014; Park et al., 2014; Kaufmann et al., 2019). One of the best studied candidate drugs is Insulin-like growth factor 1 (IGF-1).

Insulin-like growth factor 1 is a growth factor and signalling molecule that is involved in growth, maturation, and ageing. In the CNS, IGF-1 plays a role in developmental and mature brain synaptic plasticity (Dyer et al., 2016). IGF-1’s role in neuronal development and function presents multiple similarities to that of BDNF; however, the latter signalling molecule has limited therapeutic potential because of its inability to cross the blood-brain barrier. More recent evidence suggests that IGF-1 signalling is implicated in metabolic, homeostatic processes, which underlie synaptic plasticity and are disrupted in RTT (De Felice et al., 2014; Banerjee et al., 2016; Gazit et al., 2016; Neul et al., 2020; Crivellari et al., 2021). IGF-1 is naturally cleaved by proteases into the small tripeptide Glycine-Proline-Glutamic acid (GPE) and the larger Des (1–3) IGF-1 peptide. GPE has neuroprotective properties through different modulatory processes to those of IGF-1 (Guan and Gluckman, 2009). Both full-length IGF-1 and GPE have been shown to ameliorate features of relevance to RTT in a genetic mouse model of the disorder (Tropea et al., 2009; Castro et al., 2014). These encouraging results have led to clinical trials in RTT using either a recombinant human form of IGF-1 (i.e., rhIGF-1, mecasermin) or a modified GPE (i.e., trofinetide). Both mecasermin and trofinetide have shown safety and tolerability and initial evidence of efficacy (Pini et al., 2012, Pini et al., 2016b; Khwaja et al., 2014; Glaze et al., 2017, 2019; O’Leary et al., 2018).

The first clinical trial of mecasermin, an open label phase I pharmacokinetic and exploratory efficacy study, demonstrated that mecasermin reached the CNS compartment following a non-linear kinetics with greater distribution in the peripheral compartment (Khwaja et al., 2014). In terms of efficacy, several parameters showed improvements during a 20-weeks open label extension (OLE) of the pharmacokinetic segment. Improvements in measures of anxiety and mood during the OLE were associated with reversal of right frontal alpha band asymmetry on EEG, a biomarker of these behavioural abnormalities. Cardiorespiratory measures showed that apnoea, a characteristic and severe breathing abnormality in RTT, also improved markedly. Since these assessments were carried out by plethysmography, an objective methodology, these were considered the most meaningful clinical findings of the study (Khwaja et al., 2014). A follow up larger randomised placebo-controlled phase II trial, did not replicate these findings. However, secondary endpoints measuring stereotypic behaviour and social communication demonstrated significant improvements (O’Leary et al., 2018). One of several possible explanations for the discrepancy between the two mecasermin studies is that severity of breathing abnormality, specifically a minimum apnoea index, was not part of the inclusion criteria. This resulted in that only 14 out of 30 participants in the phase II trial presented at baseline an apnoea index high enough to demonstrate treatment efficacy.

The failure in consistently demonstrating mecasermin’s efficacy in children with RTT, in conjunction with the continuous interest in trofinetide - now reporting encouraging results following adult and paediatric trials (Glaze et al., 2017, 2019) - emphasises the importance of further characterising the mechanisms underlying therapeutic responses to IGF-1-related compounds and the need for identifying biomarkers linked to clinical improvements. The present study aimed at delineating molecular profiles associated with therapeutic responses to mecasermin in children with RTT, on the basis of data from the mecasermin phase I trial. Because this trial included two periods of drug administration and their intervening washout period, it offered the possibility of evaluating the dynamics of gene expression in response to mecasermin. For this purpose, we analysed RNA profiles on whole blood samples and correlated them with apnoea responder status (Khwaja et al., 2014). We chose the latter as measure of efficacy because of its objective nature. A link between two sets of objective parameters would provide stronger evidence for molecular factors underlying clinical responses to mecasermin and, probably, also other IGF-1-related compounds. In terms of the use of RNA profiles as biomarkers, we contemplated both that RNA profiles could serve as predictors of response (i.e., baseline levels) or as surrogate endpoints (i.e., change in levels between baseline and end of treatment). Whole blood is particularly useful in this respect due to availability and accessibility of the tissue. *MECP2* is considered to be widely expressed in peripheral tissues, where it has even been shown to contribute to certain RTT symptoms, including hypoactivity, exercise fatigue, and bone abnormalities (Song et al., 2014; Ross et al., 2016). Moreover, a recent study of *Mecp2* mutant mice revealed that some genes differentially expressed in blood are also altered in brain (Sanfeliu et al., 2019). These factors indicate that whole blood is a particularly relevant sample source for the aims of our study.

We found that individuals with RTT, severe breathing abnormalities and positive response to mecasermin, as shown by an improved apnoea index, had molecular signatures of relevance to their phenotype and treatment that can be distinguished before drug administration and, to a lesser extent, at later timepoints. This finding could assist in the design and analysis of future trials with mecasermin and other IGF-1-related compounds.

## Materials and Methods

### Outcome Measures and Definition of Responder

**FIGURE 1:**
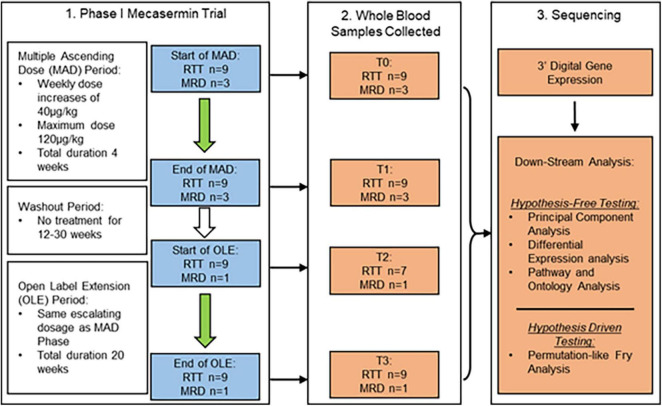
Study design and dosing schedule of phase I mecasermin trial in RTT. All participants (*n* = 12) were included in the multiple ascending dose (MAD) period while only those with RTT (*n* = 9; *n* = 7 at T2) and one with MRD progressed onto the open label extension (OLE) component. Participants were administered mecasermin twice daily by subcutaneous injection. Time points when blood sampling was performed are denoted T0 (start of MAD), T1 (end of MAD), T2 (start of OLE), and T3 (end of OLE). Whole blood samples were used for 3′-Digital Gene Expression (3′-DGE) sequencing.

Multiple outcome measures were used for evaluating efficacy. They included a wide range of clinician- and caregiver-rated neurobehavioural assessments, several of them developed or adapted for RTT [Clinical Global Impression scales (CGI-S, CGI-I), Parent-Targetted Symptom Visual Analogue Scale (PTSVAS), Mullen Scales of Early Learning (MSEL), the Clinical Severity Scale (CSS), the Motor–Behavioural Assessment (MBA), the Rett Syndrome Behaviour Questionnaire (RSBQ), and the Anxiety, Depression, and Mood Scale (ADAMS)], and automated cardiorespiratory measures. The latter consisted of time synchronised chest respiratory inductive plethysmography and electrocardiography. While neurobehavioural assessments were performed only during the OLE, cardiorespiratory measures were collected throughout the trial. Using a wireless data-acquisition plethysmography device (BioRadio, Great Lakes Neurotechnologies, Independence, OH, United States), breathing abnormality profiles with a focus on breath holding were determined for all participants. Clinically significant apnoea, which was defined as apnoeic episodes >10 s in length, was present in five participants. Apnoea was graded as moderate–severe when these apnoeic episodes were > 5 per hour, a pattern present in 4 participants at the beginning of the study, before the multiple ascending dose (MAD)/pharmacokinetics period (Figure 1 and Table 1). All these four participants experienced a decrease in apnoea severity to mild (<5 episodes per hour) by the end of the OLE (Table 1). Details on outcome measures can be found in Khwaja et al. (2014). For the purpose of evaluating therapeutic responses, Apnoea Responder was defined as a participant who had a decrease in apnoea frequency >50% or a reduction to ≤5 apnoeic episodes per hour (the four participants mentioned above). By default, the five participants who did not fulfil the criteria for Responder (R) were assigned to the Mecasermin Study Reference (MSR) group.

**TABLE 1:** Apnoea index profiles of participants with classic Rett syndrome (RTT).

Participants ID	Breathing phenotype at baseline	Apnoea index Start of MAD	Apnoea index End of MAD	Apnoea index Start of OLE	Apnoea index End of OLE
#1 MSR	None	0	1	1	1
#4 MSR	BH, HV, and AE	0	0	2	0
#5 MSR	BH and HV	0	0	0	0
#6 MSR	AE	2	1	3	1
#7 R	BH and AE	8	2	3	2
#8 MSR	AE	0	0	0	0
#9 R	BH and AE	7	4	5	3
#11 R	BH, AE, and Cyanosis	60	30	22	18
#12 R	BH. AE, and Cyanosis	14	8	6	2

*BH, breath holding; HV, hyperventilation; AE, air expulsion; R, responder group; MSR, Mecasermin Study Reference group.*

Of the total 12 clinical trial participants, nine had a diagnosis of classic RTT (participants #1, #4, #5, #6, #7, #8, #9, #11, and #12) while three participants had a diagnosis of *MECP2* related disorder (MRD) (participants #2, #3, and #10). Participants with MRD are characterised by having pathogenic *MECP2* mutations, some of them also identified in individuals with RTT, but do not display a clinical presentation compatible with either typical or atypical RTT. They were included in the trial (Khwaja et al., 2014) in order to determine whether treatment with mecasermin could be effective in most phenotypes associated with *MECP2* mutations. However, since participants with MRD did not present a breathing phenotype and were not included in the OLE period, their samples were analysed in this molecular study only as pre-treatment reference data. *MECP2* mutations of each participant with classic RTT are described in Table 2. All RTT participants were included in both the MAD and OLE treatment periods, but only 1 MRD participant (participant #10) was included in the OLE phase (see Figure 1). Mutations in participants with RTT were classified according to their profile of severity as severe (R168X, R255X, deletions and insertions), intermediate (T158M) or mild (other point mutations), following the report by Cuddapah et al. (2014).

**TABLE 2:** Methyl-CpG binding protein 2 (*MECP2*) mutation profiles of participants with classic RTT.

Participant ID, Analysis group	Mutation (nucleotide nomenclature)	Mutation (amino acid nomenclature)	Mutation location (MeCP2 domain)	Mutation type	Mutation severity profile
#1, MSR	c.538C > T	R168X	ID, TRD	Non-sense	Severe
#4, MSR	c.790_808del119	–	TRD-NLS	Deletion	Severe
#5, MSR	Deletion of Exon 3 and 4 (min 6.0kb – max 7.1kb)	–	Multiple	Deletion	Severe
#6, MSR	c.1159_1273del114	–	C-Term	Frameshift, Insertion or Deletion	Mild
#7, R	c.763C > T	R255X	TRD	Non-sense	Severe
#8, MSR	c.763C > T	R255X	TRD	Non-sense	Severe
#9, R	c.473C > T	T158M	MBD	Missense	Intermediate
#11, R	Deletion Exon 1 and 2	–	Start codon	Deletion	ND
#12, R	c.965C > T	R322L	C-Term	Missense	Mild

*MSR, Mecasermin Study Reference group; R, responder group; ID, interdomain; TRD, transcriptional repression domain; NLS, nuclear localisation signal; C-term, carboxy-terminus; MBD, methyl-binding domain; ND, not yet determined.*

### RNA Collection and Sequencing

RNA from whole blood samples was collected and extracted using PAXgene Blood RNA tubes (BD Biosciences, Radnor, PA, United States), and analysed by 3′-Digital Gene Expression (3′-DGE). Sequencing was performed at the Massachusetts Institute of Technology (MIT)’s BioMicro Center, an integrated genomics core facility. 3′-DGE was adapted from Soumillon et al. (2014) using a tag-based transcriptome sequencing method, which provides cost-effective means of generating expression data for characterising major patterns in heterogeneous samples (Soumillon et al., 2014). The sequencing read data was then combined with barcode information in a FASTQ format and mapped onto the Hg19 reference sequence using BWA. Per-gene count quantification was conducted with the End Sequence Analysis Toolkit (ESAT) for downstream differential gene expression analysis (Derr et al., 2016). Raw data will be available at the public repository Gene Expression Omnibus (GSE198856). Samples from two participant (participant #5 and participant #12) were not collected at T2 (beginning of OLE); these missing data were not imputed in the analyses.

### Differential Gene Expression Analyses

Differential gene expression was quantified using EdgeR, a popular software specifically designed for analysing sequencing data from small sample sizes (Robinson et al., 2009). EdgeR was operated on an R studio environment using R statistical programming language (Venables et al., 1995). EdgeR uses an empirical Bayes estimation, based on a negative binomial model, and a quasi-likelihood F test (QLFT) to determine differential expression. QLFT is the preferable choice for comparing gene expression on small samples, as it better reflects the uncertainty of estimating gene expression dispersion (i.e., variability), resulting in a lower error rate (Chen et al., 2017).

Using QLFT, we conducted two different types of analyses to examine the drug’s effect on gene expression across the trial: (1) time-point and (2) responder status comparisons. Based on sampling, there were six possible time-point comparisons (T0–T1, T0–T2, T0–T3, T1–T2, T1–T3, and T2–T3), which were conducted on all RTT sample sets (*n* = 9 at T0, T1, and T3; *n* = 7 at T2). On the other hand, responder status comparisons contrasted R (*n* = 4 at T0, T1, and T3; *n* = 3 at T2) and MSR (*n* = 5 at T0, T1, and T3; *n* = 4 at T2) groups at each of time point.

Significant gene sets identified by these comparisons were used to conduct pathway analysis using Reactome^1^ and Ontology analysis using Panther’s Gene Ontology database (GO).^2^ The gene sets were entered into these online tools filtering out any unidentified genes. Both Reactome and GO analysis use over-representation analysis to determine if a given gene set is over- or under-represented in a given pathway or ontology, with respect to a hypothetical random selection (Fabregat et al., 2018; The Gene Ontology Consortium, 2000, 2021). In both GO and Reactome analyses, significant pathways were considered those with Entities false discovery rate (FDR) values < 0.05, and the significant pathways were validated with Fry() to eliminate false positives caused by correlations between genes in the set. This function, uses operations (analogous to fractional permutations) on the gene sets to determine if a gene set was differentially expressed across randomly generated comparison sets (Wu et al., 2010; Chen et al., 2016; Muley et al., 2020; Grisaru-Tal et al., 2021). In summary, Fry(), by cross-checking the selected gene sets shuffling the data between the two compared groups, controls for false positives. This analysis was also employed to validate the pathways and ontologies that had been identified using the hypothesis-free methods. Only pathways validated with Fry() are reported in this study.

Using edgeR, two categories of analyses were performed:

(A) *Hypothesis-free testing*: We evaluated the differential expression of all the annotated genes expressed in all the participants (26,116 genes in total). This approach was used to detect changes in gene expression without any bias from previous studies or the literature. This type of analysis is, however, curtailed by the requirement of a high FDR due to the large number of tested genes. Hypothesis-free testing was conducted using the QLFT() function.

(B) *Hypothesis-driven testing*: We also used a hypothesis-driven (HD) approach for testing specific genes, based on previous research or the literature. Specifically, we tested five main classes of gene sets that have been associated to RTT pathophysiology: IGF-1 and BDNF pathways; metabolic homeostatic mechanisms, including mitochondria, protein ubiquitination, and chromatin mediated processes (Pecorelli et al., 2013); abnormal inflammatory responses (Maezawa and Jin, 2010; O’Driscoll et al., 2013; Lin et al., 2016; Zhao et al., 2017); pathways linked to the apnoea phenotype (e.g., monoamine metabolism) (Viemari et al., 2005; Toward et al., 2013; Vogelgesang et al., 2018); and autism spectrum disorder (ASD) iPSCs-IGF-1 induced genomic changes (Linker et al., 2020). To test whether these gene sets were differentially expressed across the time-point comparisons or the R vs. MSR comparisons, we used edgeR’s Fry function. Gene sets analysed with Fry were further validated by permutation analyses (Bach et al., 2020), which take into consideration control pathways with the same size of the HD gene sets and use the distribution of the *p*-value of the controls and the HD gene sets to confirm statistical significance. Only HD gene-sets with *p*-values falling into the top 5 percentile of the *p*-value distribution are reported in this study.

### Statistical Analysis and Graphing Software

Principal component analysis (PCA) was conducted using the built-in prcomp() function in R studio, by analysing sample counts at T0, applying scaling and centring of data. For graphical representations, we used the ggplot2 package, also in R studio. All the tests were corrected for multiple testing by FDR, and differences were defined as significant with a *p*-value < 0.05. Analyses examining the relationship between PCs and variables of clinical significance were performed by the non-parametric Spearman rank correlation test.

## Results

### Cohort and Trial Design

The study is regulated by Institutional Review Board protocol number 10-08-0403, and by MTA agreement #18081. The cohort in the phase I open label mecasermin trial (Khwaja et al., 2014) included 9 participants with classic RTT and 3 with *MECP2*-related disorders [MRD; non-RTT clinical presentations in individuals with *MECP2* mutations (Neul et al., 2010)]. Details about the cohort, including individual *MECP2* mutations in participants with RTT, can be found in Table 2 and in the original publication on the trial by Khwaja et al. (2014). The study consisted of two different components: a 4-week multiple ascending dose (MAD) period and a 20-week open label extension (OLE) period. The MAD and OLE periods were separated by a variable interval of 12–30 weeks. The MAD period was focussed on assessment of safety and collection of serial pharmacokinetic data. The goal of the OLE period was to extend the evaluation of mecasermin safety and to conduct a preliminary assessment of the drug’s efficacy. Both MAD and OLE periods began with a dose of 40 μg/kg, which was increased by 40 μg/kg per week to a maximum dose of 120 μg/kg. Whole blood samples were taken from the participants at four different time points denoted T0–T3 (T0 and T1, corresponding to the beginning and end of the MAD, respectively; and T2 and T3, corresponding to the beginning and end of the OLE, respectively). Of note, for participants #5 and #12 samples were not obtained at T2. At T2 and T3 (OLE period), in addition to the cardiorespiratory evaluations described below, multiple neurobehavioral assessments were also performed (details in Khwaja et al., 2014). A schematic of the dosing schedule and study design are shown in Figure 1.

### Gene Expression Profiles in Participants With Rett Syndrome and *MECP2* Related Disorder

The first treatment period, the Multiple Ascending Dosage (MAD) period, intended to determine the pharmacokinetics of mecasermin. The second treatment period, the Open Label Extension (OLE), was an additional treatment segment intending to obtain additional information on safety and preliminary data on efficacy. The MAD period included, in addition to the nine participants with classic RTT, three girls with MRD. *MECP2* mutations identified in each participant with RTT are described in Table 2.

We found no significant differential gene expression, calculated with edgeR, when comparing MRD and RTT groups at T0. Because the T1, T2, and T3 time points had only one MRD sample, participants with MRD were not considered for further analysis. Table 3 presents the changes in gene expression in the entire RTT cohort throughout the trial, by comparing the different sequential time points.

**TABLE 3:** Differentially expressed genes in the entire RTT cohort throughout the trial.

Interval	Gene	Log2 Fold Change	*p*-value	FDR
T0–T1	–	–	–	–
T0–T2	–	–	–	–
T0–T3	–	–	–	–
T1–T2	*TMEM176B*	−2.53	1.54 × 10^–11^	3.41 × 10^–07^
	*TMEM176A*	−2.72	2.61 × 10^–11^	3.41 × 10^–07^
T1–T3	–	–	–	–
T2–T3	*ERVMER34-1*	2.35	3.22 × 10^–09^	7.81 × 10^–05^
	*TMEM176B*	2.07	5.98 × 10^–09^	7.81 × 10^–05^
	*RRM2*	2.06	3.42 × 10^–06^	0.03
	*CENPF*	2.81	5.07 × 10^–06^	0.03

### Gene Expression Profiles of Responder and Mecasermin Study Reference Groups at Baseline

**FIGURE 2:**
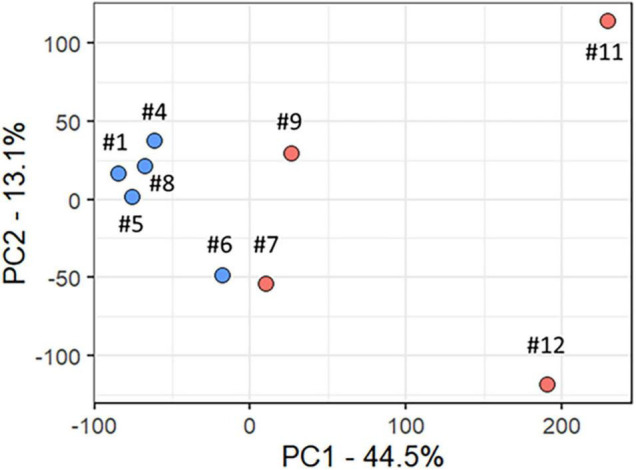
Principal component analysis of baseline transcript profiles in participants with RTT. Scatter plot of Principal Components 1 and 2 (PC1 and PC2), which accounted, respectively, for 44.5 and 13.1% of total variance. The Responder group is shown in red and the Mecasermin Study Reference group in blue. Note that participant #6 did not meet all criteria for the Responder group; however, she also showed an improvement in her apnoea index at the end of the trial.

All analyses described in this and the following sections, used only data from the participants with RTT. We used PCA to delineate the relationship between gene expression profiles at baseline (T0) in R (participants #7, #9, #11, and #12) versus MSR (participants #1, #4, #5, #6, and #8) groups. Figure 2 shows a plot of the largest principal components (PC1 and PC2) at T0 which accounted, respectively, for 44.5 and 13.1% of the variance. R (*n* = 4, red) and MSR (*n* = 5, blue) groups were divided by PC1; R to the right and MSR to the left of 0 on the PC1 axis. Participants in the MSR group were relatively close to each other with exception of one sample (participant #6), varying mainly in PC2, while the R group was more dispersed. Interestingly, although that MSR participant did not meet our stringent criteria for responder, she had a mild apnoea phenotype with episodes of the same length as those in the R group and showed an improvement in her apnoea index at the end of the OLE. As reference, the PC profile of MRD participants is intermediate between the R and MSR groups. Thus, this gene expression variance analysis showed that R and MSR groups segregated from each other before treatment with mecasermin along PC1, which is consistent with their clinical profiles and outcome. Analyses examining the relationship between gene expression profiles, measured by PC1, and variables of clinical significance, found no significant relationship between PC1 and mutation severity category (Rho = 0.52, *p* = 0.15). In contrast, we found a strong correlation between PC1 and apnoea index at baseline. When considering all the participants in the study (*n* = 9), the correlation was significant (Rho = 0.94, *p* = 0.00016). However, when considering only participants with apnoea index > 0 (*n* = 5), although the correlation was strong (Rho = 0.90), the test failed to reach significance (*p* = 0.08; Supplementary Figure 1).

### Gene Expression Profiles of Responder and Mecasermin Study Reference Groups Throughout the Trial

In order to determine how the R and MSR groups reacted differentially to IGF-1 treatment, we performed gene expression analysis at different time-points throughout the trial. We compared gene expression between the two groups at T0, T1, T2, and T3. Such comparisons revealed a large number of changes (Supplementary Table 1) with the greatest difference in gene expression observed at T0, as presented in detail in the previous section, and relatively higher overall expression in the R group with respect to the MSR group (3,693 upregulated, 221 downregulated). Similar patterns, with relatively higher expression in R versus MSR groups, were also observed at T1 (113 upregulated, 34 downregulated) and T2 (66 upregulated, 16 downregulated). However, the direction of these differences was reversed at T3 (2 upregulated, 28 downregulated). Thus, the analyses revealed decreasing differential gene expression between the R and MSR groups, from baseline to end of treatment (T0–T3), confirming the existence of different “molecular” subcohorts at the beginning of the trial. The two groups, identified by their genomic profiles, appeared to respond differently to mecasermin treatment, with the MSR group showing more changes (Table 4).

**TABLE 4:** Number of genes differentially expressed in the RTT cohort throughout the trial.

Interval	Number of genes (all RTT, *n* = 9; *n* = 7 at T2)	Number of genes (R, *n* = 4; *n* = 3 at T2)	Number of genes (MSR, *n* = 5; *n* = 4 at T2)
T0–T1	–	1 (0 ↑, 1 ↓)	13 (1 ↑, 12 ↓)
T0–T2	–	2 (0 ↑, 2 ↓)	24 (0 ↑, 24 ↓)
T0–T3	–	–	28 (16 ↑, 12 ↓)
T1–T2	2 (0 ↑, 2 ↓)	–	49 (5 ↑, 44 ↓)
T1–T3	–	–	27 (21 ↑, 6 ↓)
T2–T3	4 (4 ↑, 0 ↓)	1 (1 ↑, 0 ↓)	37 (36 ↑, 1 ↓)

*↑, upregulated,↓, downregulated.*

In order to identify pathways differentially regulated in R versus MSR groups at different periods of the trial, the list of differentially expressed genes was input into Reactome (pathway database) and Gene Ontology (GO). Results were controlled for false positives using Fry() (EdgeR). GO found significantly enriched ontologies only at T0 (all upregulated in the R group). The top 50 validated GO gene sets are depicted in Table 5, while the full list is reported in Supplementary File 2. Validated pathways in Reatcome, at different time points, are shown below in Table 6.

**TABLE 5:** Top 50 GO gene sets differentially regulated in R and MSR groups throughout the trial.

Comparison	ID	Gene ontology
RVMSR.T0	GO:0008152	Metabolic process
RVMSR.T0	GO:0051641	Cellular localisation
RVMSR.T0	GO:0022414	Reproductive process
RVMSR.T0	GO:0051252	Regulation of RNA metabolic process
RVMSR.T0	GO:0048870	Cell motility
RVMSR.T0	GO:0044237	Cellular metabolic process
RVMSR.T0	GO:0040011	Locomotion
RVMSR.T0	GO:0032446	Protein modification by small protein conjugation
RVMSR.T0	GO:0044260	Cellular macromolecule metabolic process
RVMSR.T0	GO:0098916	Anterograde trans-synaptic signalling
RVMSR.T0	GO:0050953	Sensory perception of light stimulus
RVMSR.T0	GO:0097746	Blood vessel diameter maintenance
RVMSR.T0	GO:0030198	Extracellular matrix organisation
RVMSR.T0	GO:0001704	Formation of primary germ layer
RVMSR.T0	GO:0031640	Killing of cells of another organism
RVMSR.T0	GO:0044419	biological process involved in interspecies interaction between organisms
RVMSR.T0	GO:0099536	Synaptic signalling
RVMSR.T0	GO:0007601	Visual perception
RVMSR.T0	GO:0007157	Heterophilic cell–cell adhesion via plasma membrane cell adhesion molecules
RVMSR.T0	GO:0051480	Regulation of cytosolic calcium ion concentration
RVMSR.T0	GO:0034329	Cell junction assembly
RVMSR.T0	GO:0035296	Regulation of tube diameter
RVMSR.T0	GO:0043062	Extracellular structure organisation
RVMSR.T0	GO:0050808	Synapse organisation
RVMSR.T0	GO:0099537	Trans-synaptic signalling
RVMSR.T0	GO:0061844	Antimicrobial humoural immune response mediated by antimicrobial peptide
RVMSR.T0	GO:0097485	Neuron projection guidance
RVMSR.T0	GO:0002376	Immune system process
RVMSR.T0	GO:0035150	Regulation of tube size
RVMSR.T0	GO:0007411	Axon guidance
RVMSR.T0	GO:0007155	Cell adhesion
RVMSR.T0	GO:0008015	Blood circulation
RVMSR.T0	GO:0048871	Multicellular organismal homeostasis
RVMSR.T0	GO:0003018	Vascular process in circulatory system
RVMSR.T0	GO:1903522	Regulation of blood circulation
RVMSR.T0	GO:0007409	Axonogenesis
RVMSR.T0	GO:0007268	Chemical synaptic transmission
RVMSR.T0	GO:0000902	Cell morphogenesis
RVMSR.T0	GO:0007369	Gastrulation
RVMSR.T0	GO:0001944	Vasculature development
RVMSR.T0	GO:0000904	Cell morphogenesis involved in differentiation
RVMSR.T0	GO:0098609	Cell–cell adhesion
RVMSR.T0	GO:1903034	Regulation of response to wounding
RVMSR.T0	GO:0003013	Circulatory system process
RVMSR.T0	GO:0048667	Cell morphogenesis involved in neuron differentiation
RVMSR.T0	GO:0048646	Anatomical structure formation involved in morphogenesis
RVMSR.T0	GO:0061564	Axon development
RVMSR.T0	GO:0072359	Circulatory system development
RVMSR.T0	GO:0001568	Blood vessel development
RVMSR.T0	GO:0048812	Neuron projection morphogenesis

*All gene sets are upregulated in the R group with respect to the MSR group.*

*RVMSR, Responder group versus Mecasermin Study Reference group.*

**TABLE 6:** Validated differentially regulated Reactome pathways in R and MSR groups throughout the trial.

Comparison	ID	Reactome pathways	Direction	*p*-Value	FDR
RVMSR.T0	R-HSA-1474228	Degradation of the extracellular matrix	Up	0.000867	0.001536
RVMSR.T0	R-HSA-1474244	Extracellular matrix organisation	Up	0.001152	0.001536
RVMSR.T0	R-HSA-6805567	Keratinisation	Up	0.001545	0.001536
RVMSR.T0	R-HSA-1266738	Developmental Biology	Up	0.000997	0.001536
RVMSR.T2	R-HSA-1462054	Alpha-Defensins	Up	0.006901	0.001536
RVMSR.T2	R-HSA-1474228	Degradation of the extracellular matrix	Up	0.00809	0.001536
RVMSR.T2	R-HSA-1474244	Extracellular matrix organisation	Up	0.015394	0.001536
RVMSR.T2	R-HSA-8939242	RUNX1 regulates transcription of genes involved in differentiation of keratinocyte	Up	0.011898	0.001536

*The full list of validated Reactome gene sets is included in Supplementary Table 2.*

*RVMSR, Responder group versus Mecasermin Study Reference group.*

### Preliminary Assessment of Mechanisms Underlying Response to Mecasermin in Rett Syndrome: Hypothesis-Free Analysis

In order to ascertain mechanisms underlying the response to mecasermin in RTT, we examined differential gene expression in the entire RTT cohort (*n* = 9; *n* = 7 at T2) at different intervals (T0–T1, T0–T2, T0–T3, T1–T2, T1–T3, and T2–T3). There were no significant differences between T0 and T1 (MAD period), T0 and T2, T0 and T3 (entire trial), or T1 and T3. During the off-treatment period (T1–T2), two related genes *TMEM176A* and *TMEM176B* showed a reduction in levels (Table 3). In the interval corresponding to the OLE period (T2–T3), we found 4 differentially expressed genes (i.e., increased expression): *ERVMER34-1*, *RRM2*, *CENPF*, and the abovementioned *TMEM176B*. Thus, mecasermin treatment induced limited changes in gene expression that were mainly present during the OLE period (Supplementary Material), most likely due to the heterogeneity of the population. Therefore, we included separate comparisons of gene expression patterns across the different study intervals in the R (*n* = 4; *n* = 3 at T2) and MSR (*n* = 5; *n* = 4 at T2) groups.

The R group showed only two differentially expressed genes across all study intervals: *HLA-DRB5* and *SMCR5. HLA-DRB5* encodes the major histocompatibility complex class II DRβ5, *SMCR5* is the non-coding *Smith-Magenis Syndrome Chromosome Region Candidate 5* gene. In the R group, *HLA-DRB5* decreased significantly from T0 to T1 (MAD period) and from T0 to T2, but it increased significantly during the OLE (T2–T3). In contrast, in the MSR group *HLA-DRB5* expression decreased only during the OLE (T2–T3).

Many of the genes differentially expressed along the study in the MSR group have roles in the immune system. Among these immune function genes are the *TMEM176* genes, which are associated with maintenance of dendritic cell immaturity (Condamine et al., 2010). *TMEM176A* is differentially expressed in the MSR group between T1 and T2 (*p* < 0.01), while *TMEM176B* is differentially expressed between T1 and T2 (*p* < 0.01) and T2 and T3 (*p* < 0.02). Fold change levels are reported in Supplementary Table 1. The MSR group also showed increases in several defensin-α genes. Defensins are antimicrobial and cytotoxic peptides involved in host defence, which are stored in granules (azurophils). During phagocytosis, these granules fuse into phagocytic vacuoles and contribute to antimicrobial response (Ganz, 2003). The increases in defensin gene expression mainly represent changes during the OLE (T2 to T3), but also between T0 and T3 and T1 and T3 (all *p*-values < 0.001; for detailed fold changes see Supplementary Table 1).

Overall, the differential gene expression analyses between different periods of the trial revealed a significant change only in the MSR group. At all intervals, we found several differentially expressed genes: 13 genes in T0–T1, 24 genes in T0–T2, 28 genes in T0–T3, 49 genes in T1–T2, 27 genes in T1–T3, and 37 genes in T2–T3. Table 4 summarises interval comparisons in all three groups under analysis: all participants with RTT, R group, and MSR group. A full list of results is included in Supplementary Table 1.

All the differentially expressed genes identified in the entire cohort were also found in the MSR group, suggesting that the differences in the entire cohort were mainly driven by the former. Therefore, all subsequent analyses of differential gene expression were carried out separately in the R and MSR groups. We used the differentially expressed genes identified in these two groups to perform pathway and ontology analyses using Reactome and GO. The significant results from these analyses were then validated using the edgeR’s Fry function. The validated results, all in the MSR group, are: Mitotic Cell Cycle Process (GO:1903047) upregulated at T3 versus T2, Non-sense mediated Decay (NMD) (R-HSA-927802), downregulated at T2 versus T1, and NMD enhanced by exon Junction Complex (R-HSA-975957) upregulated at T2 versus T1.

### Preliminary Assessment of Mechanisms Underlying Response to Mecasermin in Rett Syndrome: Hypothesis-Driven Testing

We then tested if mechanisms previously associated to RTT pathogenesis were different between R and MSR groups, and if they were modulated by the administration of Mecasermin. We retrieved the corresponding gene sets in GO and then tested the hypothesis using permutation analyses in R (edgeR’s Fry function). This analysis showed significant results in R versus MSR comparisons, at T0, T1, and T2.

All the results of hypothesis-driven testing are included in Table 7. The analysis reveals that most of the gene sets were differentially expressed between R and MSR groups at T0, except for the BDNF receptor signalling pathway. At T1, which corresponded to the end of the MAD period, the IGF-1 receptor signalling pathway was differentially expressed. At T2, a number of signalling pathways were differentially expressed including PI3K, BDNF receptor, dopamine receptor, and serotonin receptor. At T3, there were no significantly different gene sets when R and MSR groups were compared. Results of R vs. MSR group analyses are shown in Table 7.

**TABLE 7:** Pathways evaluated in hypothesis-driven analysis.

Comparison	Gene ontology	ID	Direction	*p*-Value	FDR
RVMSR T0	BDNF receptor signalling pathway	GO.0031547	Up	0.0212	0.023388
RVMSR T0	Serotonin receptor signalling pathway	GO.0007210	Up	0.0016	0.005957
RVMSR T0	Dopamine receptor signalling pathway	GO.0007212	Up	0.0002	0.003271
RVMSR T0	Response to catecholamine	GO.0071869	Up	0.0018	0.005957
RVMSR T0	Linker et al. ASD_Chronic	ASD_Chronic	Up	0.0028	0.006034
RVMSR T0	MAPK cascade	GO.0000165	Up	0.0010	0.005957
RVMSR T0	Phosphatidylinositol 3-kinase signalling	GO.0014065	Up	0.0036	0.006208
RVMSR T0	Inflammatory response	GO.0006954	Up	0.0025	0.005957
RVMSR T0	Protein ubiquitination	GO.0016567	Up	0.0024	0.005957
RVMSR T0	Linker et al. Control_Acute	Control_Acute	Up	0.0040	0.006208
RVMSR T0	Reactive oxygen species metabolic process	GO.0072593	Up	0.0024	0.005957
RVMSR T0	Chromatin organization	GO.0006325	Up	0.0083	0.010816
RVMSR T0	Linker et al. ASD_Acute	ASD_Acute	Up	0.0220	0.023388
RVMSR T1	Insulin-like growth factor receptor signalling pathway	GO.0048009	Up	0.0005	0.008814
RVMSR T2	Phosphatidylinositol 3-kinase signalling	GO.0014065	Up	0.0004	0.003133
RVMSR T2	Response to catecholamine	GO.0071869	Up	0.0000	0.00052
RVMSR T2	BDNF receptor signalling pathway	GO.0031547	Up	0.0065	0.022009
RVMSR T2	Dopamine receptor signalling pathway	GO.0007212	Up	0.0043	0.02021
RVMSR T2	Serotonin receptor signalling pathway	GO.0007210	Up	0.0088	0.024885
RVMSR T2	Reactive oxygen species metabolic process	GO.0072593	Up	0.0048	0.02021
R T1T2	insulin-like growth factor receptor signalling pathway	GO.0048009	Up	0.001056	0.017946
MSR T1T2	Linker et al. ASD_Chronic	ASD_Chronic	Down	6.93E-09	1.18E-07

*Gene sets were considered differentially expressed with a FDR < 0.05.*

*RVMSR, Responder group versus Mecasermin Study Reference group.*

The same sets of genes were then examined within each group (R and MSR) at different intervals. We found that in the R group only the IGF-1 receptor signalling pathway was differentially expressed from T1 to T2, while in the MSR group Response to Chronic IGF-1 treatment in ASD iPSCs was significantly different between T1 and T2 (Table 7).

**FIGURE 3:**
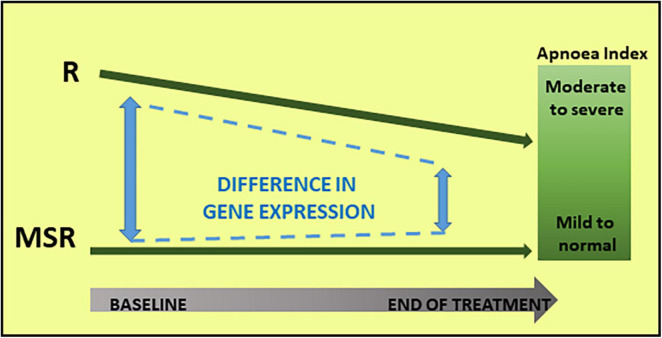
Molecular and phenotypic changes in Responder (R) and Mecasermin Study Reference (MSR) groups throughout the study. Based on their breathing phenotype, the cohort was divided into two groups: Responder and MSR groups. The R group, including participants with moderate to severe breath holding phenotype, responded to mecasermin administration by decreasing their apnoea index. Because of their virtual lack of breath holding phenotype, those in the MSR group experienced minimal changes. In parallel to these changes in breathing phenotype in the R group, there was a decrease in group differences in transcript profiles (i.e., significant at baseline) throughout the study.

In summary, both hypothesis-driven and hypothesis-free analyses demonstrated that the RTT cohort was not homogeneous at baseline, and in its molecular response to mecasermin treatment. Figure 3 summarises the results of the gene expression analyses in relationship with changes in the apnoea index.

## Discussion

### Molecular Biomarkers and Response to Mecasermin Treatment

The present study aimed at identifying RNA profiles associated with therapeutic responses to mecasermin in children with RTT. For this purpose, we used samples from a mecasermin phase I trial, which explored in a preliminary fashion clinical response to the compound in a study including two periods of drug administration. Of the two positive clinical endpoints, we selected breathing abnormalities because it included an objective measure: plethysmography. We defined as Responder to the drug an individual who had moderate to severe apnoea index at baseline, and significantly improved breath holding (i.e., apnoea index) based on plethysmographic evaluations. We then compared the gene expression profiles of Responders with the rest of the cohort, which we termed MSR group, throughout the trial. We also evaluated changes within each of the two groups. Although not definitive because of the lack of a non-responder group, our analyses showing differences between R and MSR groups in gene expression that included IGF-1- and breathing phenotype-related genes at baseline suggest that RNA profiles may be able to identify individuals with RTT more likely to respond to IGF-1-like compounds.

Comparisons between T0 and T1 (first drug administration), T1–T2 (washout drug-free period), T2–T3 (second drug administration), and T0–T3 (entire trial), allowed examination of baseline gene expression and its changes in response to single and repeated mecasermin exposure. Comparisons at baseline between classic RTT and MRD groups, both included in the original study (Khwaja et al., 2014), revealed similar gene expression profiles. PC profiles placed MRD participants between the R and MSR RTT groups, as expected from patients with some RTT features but no apnoea as reported (Khwaja et al., 2014). Thus, molecular phenotypical profiles before mecasermin administration were in general correspondence with clinical phenotypes. Since the MRD group was not included in the OLE period, we focussed our gene expression analyses on the classic RTT cohort. These analyses demonstrated marked pre-treatment R versus MSR differences that diminished over time. Changes in the MSR group between T0 and T2 indicate that their gene expression profiles were modified by mecasermin treatment. The latter hypothesis is supported by the clinical changes reported by Pini et al. (2014) in a single-case study, where the authors conducted two periods of mecasermin administration (6 and 4 months, respectively) separated by a washout period of 2 years. Both administration cycles led to moderate decreases in impairments (e.g., hand wringing, bruxism, apnoea) and increases in abilities (e.g., reaching, pointing, gesturing). However, improvements were not maintained between treatment cycles and outcome profiles differed between cycles. These results support dynamic and partially compensatory responses after mecasermin administration, which could be reflected in gene expression profiles.

The main differences between R and MSR groups were observed at baseline (T0) while the main changes during the course of the trial occurred between the end of the MAD period and the beginning of the OLE period (T1–T2) and throughout the OLE (T2–T3), predominantly in the MSR group. Genes found to be differentially expressed regulate cell cycle processes and, in particular, immune responses (e.g., *TMEM176A*, *TMEM176B*). These were discrete changes of variable direction, suggesting both intrinsic RTT pathogenetic processes as well as the effects of the intervention under study.

### Gene Expression Profiles of Responders to Mecasermin in Rett Syndrome

Analyses of baseline (T0) transcript profiles from R and MSR groups revealed a clear separation between groups, as evidenced by a single principal component accounting for almost half of the variance in overall gene expression. The differences represented relatively higher expression in the R group with respect to the MSR group (i.e., 3,693 upregulated, 221 downregulated), a pattern that continued but markedly decreased in magnitude throughout the trial (Figure 3). Relevance of these gene expression patterns is supported by the significant correlation between principal components and apnoea index at baseline. Thus, the profile and dynamics of the differential transcript profiles seems to reflect an IGF-1 signalling “favourable” status at baseline in the R group, which facilitated selective cellular responses to IGF-1 administration exemplified by the decrease in *HLA-DRB5* expression. On the other hand, mecasermin administration in the MSR group led to multiple presumably adaptive molecular changes throughout the study. These hypotheses are supported, first, by the unbiased, hypothesis-free analysis of pathways and mechanisms that revealed baseline differences in the expression of genes regulating vascular dynamics (i.e., vasoconstriction, vascular permeability), extracellular matrix, or inflammatory/immune responses. These functions are in line with the main phenotype targetted by mecasermin, namely breathing abnormalities, as well as with peripheral vasomotor disturbances commonly observed in individuals with RTT. The fact that the participant in the MSR group with a milder breathing phenotype and improvement had a gene expression profile similar to the R group, underscores the relationship between transcript patterns and clinical profiles and outcomes. Hypothesis-driven analyses discussed below provided additional support to the notion that the identified changes in gene expression were in response to IGF-1 administration.

### Mechanisms Underlying Positive Response to Mecasermin in Rett Syndrome

To get additional insight into the mechanisms underlying positive response to mecasermin, we examined gene expression dynamics throughout the study using hypothesis-driven analyses. We selected pathways and mechanisms that have been implicated in the pathophysiology of RTT, such as MAPK and PI3K signalling (Tropea et al., 2009; Mellios et al., 2014), BDNF (Zhou et al., 2006), metabolic abnormalities in RTT such as mitochondrial dysfunction (Shulyakova et al., 2017; Shovlin and Tropea, 2018), and immunological function (Maezawa and Jin, 2010; Shovlin and Tropea, 2018). We also considered pathways associated to monoamine modulation (Viemari et al., 2005; Toward et al., 2013; Pini et al., 2016a; Vogelgesang et al., 2018) and IGF-1 signalling (Linker et al., 2020) since these are relevant to the breathing phenotype and its treatment with mecasermin. These analyses confirmed the involvement of inflammatory and immune responses, but also revealed differences in monoamine- and metabolism/homeostasis-related genes. Underscoring mecasermin’s mechanism of action, differential gene expression dynamics between the R and MSR groups demonstrated distinct transcript profiles related to IGF-1 signalling. Indeed, the main differences between the R and MSR groups at T0, involved the BDNF cellular pathway as well as previously reported gene expression changes in response to IGF-1 treatment in ASD iPSCs. As in the hypothesis-free analyses, most differences between the R and MSR groups were found at T0. Nonetheless, IGF-1, BDNF, and apnoea-related genes were also differential at later time points suggesting continuous action of IGF-1 on target pathways. This molecular dynamics resembles the effects of mecasermin on brain activity in participants with RTT, as previously reported by us (Keogh et al., 2020). The reason for the MSR group’s greater changes in gene expression, in response to mecasermin, is unknown and deserves further examination since these molecular changes may disclose key events associated with response to IGF-1 and related compounds.

### Potential of RNA Profiles as Molecular Signatures of Response to Mecasermin and Related Compounds

This preliminary study supports the potential of gene expression profiles as biomarkers in RTT drug trials. Although our data only revealed gene expression patterns in participants with severe breathing phenotype who also improved after mecasermin administration, and no treatment response comparison group (i.e., severe apnoea without improvement) was available, the nature and evolution of the expression profiles (i.e., correlated with apnoea index at baseline, higher expression of IGF-1 signalling and monoamine modulation genes) suggest that they were treatment related. This and the fact that other informative genes in this study are in line with our current knowledge of RTT pathogenesis (e.g., immune and metabolic mechanisms) underscore the relevance of the findings. Nonetheless, follow up investigations need to address response to treatment more directly. Ideally, studies with larger samples or other IGF-1 related drugs following the course of clinical responses will elucidate whether RNA profiles could become surrogate endpoints, and will provide additional validation of the reported results (i.e., qPCR).

Our findings encourage similar assessments for other drugs under preclinical and clinical investigation in RTT. While other aspects of study design, including dosage, length, and endpoints, continue to be critical for the successful outcome of drug trials in RTT, cohort selection for all candidate treatments for RTT could be improved by molecular profiling. Whether the present data will lead to a re-examination of the therapeutic potential of IGF-1 treatment will depend on follow up supportive studies. Nonetheless, ongoing RTT studies with trofinetide may benefit of the reported data.

Although the present study used an objective measure of clinical outcome, breathing patterns by plethysmography, we acknowledge several limitations. These included small sample size, wide age range, and the limited nature of the molecular investigations. Indeed, proteomics or metabolomics studies could provide additional insights into the molecular mechanisms associated with clinical outcomes. Another limitation is the use of whole blood for RNA analysis, which could be influenced by the individual’s inflammatory/immunological status and its associated variability in cell types. Analyses of RNA expression in different cell types could have been more informative, but they were not feasible in the present study. Nonetheless, we consider the reported data the first step for identifying blood-based biomarkers in drug trials of IGF-1-related compounds in RTT. Future investigations will ideally assess the correlation between blood biomarkers with brain activity and other biomarkers, as well as with a wider range of clinical endpoints. Preclinical studies in animal models will be helpful for studying the correlation between changes in candidate biomarkers and other neurologic parameters that are also measurable in humans, such as motor function and sensory processing (e.g., prepulse inhibition of the startle response) (Kaufmann et al., 2019).

## Data Availability Statement

The datasets presented in this study can be found in online repositories. The names of the repository/repositories and accession number(s) can be found below: National Center for Biotechnology Information (NCBI) BioProject database under accession number GSE198856.

## Ethics Statement

The studies involving human participants were reviewed and approved by the Boston Children’s Hospital Institutional Review Board (IRB-10-08-0403). Written informed consent to participate in this study was provided by the participants’ legal guardian/next of kin.

## Author Contributions

SS analysed the data, prepared the figures and tables, and contributed to the preparation of the manuscript. CD performed some experiments and contributed to the discussion of the data. LS contributed to data collection. SB contributed to data analysis. MSa contributed to data collection, discussion of the results, and preparation of the manuscript. MSu contributed to the discussion of the data and the preparation of the manuscript. WK contributed to the planning of the experiment, collection of the data, and preparation of the manuscript. DT contributed to the planning of the experiment, data analysis, and preparation of the manuscript. All authors contributed to the article and approved the submitted version.

## Conflict of Interest

WK was the Chief Scientific Officer of Anavex Life Sciences. The remaining authors declare that the research was conducted in the absence of any commercial or financial relationships that could be construed as a potential conflict of interest.

## Publisher’s Note

All claims expressed in this article are solely those of the authors and do not necessarily represent those of their affiliated organizations, or those of the publisher, the editors and the reviewers. Any product that may be evaluated in this article, or claim that may be made by its manufacturer, is not guaranteed or endorsed by the publisher.

## Footnotes

1 https://reactome.org/

2
http://geneontology.org/
