# Supplementary figures and images for: Molecular Signatures of Response to Mecasermin in Children With Rett Syndrome
Source: Front Neurosci. 2022 May 31;16:868008. doi: 10.3389/fnins.2022.868008 (PMC9197456; doi:10.3389/fnins.2022.868008)

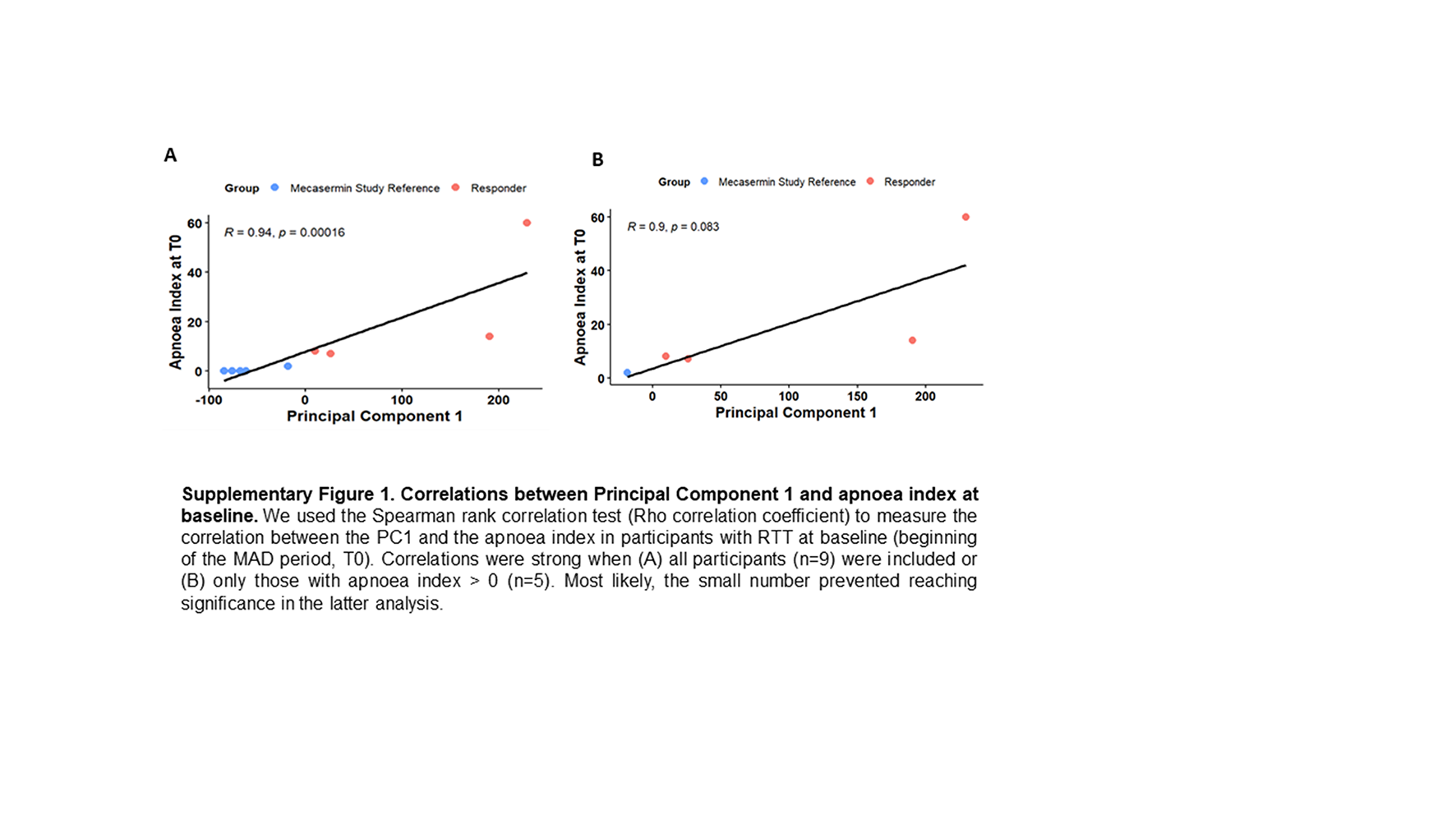

Supplement: Supplementary file 3 [file Image_1.TIF]
